# Searching for a Needle in a Haystack: Cas9-Targeted Nanopore Sequencing and DNA Methylation Profiling of Full-Length Glutenin Genes in a Big Cereal Genome

**DOI:** 10.3390/plants11010005

**Published:** 2021-12-21

**Authors:** Ilya Kirov, Ekaterina Polkhovskaya, Maxim Dudnikov, Pavel Merkulov, Anastasia Vlasova, Gennady Karlov, Alexander Soloviev

**Affiliations:** 1 Laboratory of Marker-Assisted and Genomic Selection of Plants, All-Russia Research Institute of Agricultural Biotechnology, Timiryazevskaya Str. 42, 127550 Moscow, Russia; eynzeynkreyn@gmail.com (E.P.); max.dudnikov.07@gmail.com (M.D.); paulmerkulov97@gmail.com (P.M.); vlasova.nactia@yandex.ru (A.V.); karlovg@gmail.com (G.K.); A.Soloviev70@gmail.com (A.S.); 2 Kurchatov Genomics Center of ARRIAB, All-Russia Research Institute of Agricultural Biotechnology, Timiryazevskaya Str. 42, 127550 Moscow, Russia; 3 N.V. Tsitsin Main Botanical Garden of the Russian Academy of Sciences, Botanicheskaya Str. 4, 127276 Moscow, Russia

**Keywords:** nanopore sequencing, Cas9-enrichment, triticale, glutenin genes, DNA methylation

## Abstract

Sequencing and epigenetic profiling of target genes in plants are important tasks with various applications ranging from marker design for plant breeding to the study of gene expression regulation. This is particularly interesting for plants with big genome size for which whole-genome sequencing can be time-consuming and costly. In this study, we asked whether recently proposed Cas9-targeted nanopore sequencing (nCATS) is efficient for target gene sequencing for plant species with big genome size. We applied nCATS to sequence the full-length glutenin genes (*Glu-1Ax*, *Glu-1Bx* and *Glu-1By*) and their promoters in hexaploid triticale (X Triticosecale, AABBRR, genome size is 24 Gb). We showed that while the target gene enrichment *per se* was quite high for the three glutenin genes (up to 645×), the sequencing depth that was achieved from two MinION flowcells was relatively low (5–17×). However, this sequencing depth was sufficient for various tasks including detection of InDels and single-nucleotide variations (SNPs), read phasing and methylation profiling. Using nCATS, we uncovered SNP and InDel variation of full-length glutenin genes providing useful information for marker design and deciphering of variation of individual *Glu-1By* alleles. Moreover, we demonstrated that glutenin genes possess a ‘gene-body’ methylation epigenetic profile with hypermethylated CDS part and hypomethylated promoter region. The obtained information raised an interesting question on the role of gene-body methylation in glutenin gene expression regulation. Taken together, our work disclosures the potential of the nCATS approach for sequencing of target genes in plants with big genome size.

## 1. Introduction

Target gene sequencing (TGSeq) is a set of different approaches for sequencing of specific genes without the application of whole-genome sequencing (WGS) which is an expensive alternative. It is worth noting that for species with big genome size including wheat, onion and triticale TGSeq is an optimal method because of the high price of whole-genome sequencing and difficulties in genome assembly. Yet (allo)polyploidy adds another layer of complexity for sequencing individual genes and interpreting the results.

Several approaches have been used for TGSeq including Sanger sequencing, target gene enrichment strategies with subsequent short-read sequencing and long-read sequencing. Sanger sequencing of target genes is a method of choice for end-to-end sequencing of short genes (below 1 Kb) while sequencing of longer genes requires amplification of a set of overlapping fragments. Direct Sanger sequencing of PCR products amplified from a gene can be challenging if multiple alleles are present after PCR. Several short-read based techniques have been developed and successfully used for TGSeq (reviewed by [1]). However, short-read sequencing suffers from mapping issues to repeating and low complexity regions as well as assembly errors if de novo gene assembly is used.

To deal with the main disadvantages of short-read data long-read sequencing combined with enrichment steps were developed. Very recently Xdrop method was proposed to carry out enrichment of DNA samples by target genomic fragments followed by long-read or short-read sequencing [2]. Xdrop is a very promising technology, but it needs special equipment, and it relies on DNA amplification steps that can introduce some biases and artefacts. Another long-read based approach for TGSeq called CATCH (Cas9-assisted targeting of chromosome segments) was introduced by Gabrieli et al. [3]. CATCH implements Cas9-mediated cleavage of target genome region with subsequent purification and amplification of cleaved fragments and nanopore sequencing. By this method, authors sequenced 200 Kb genomic region with 80 Kb BRCA1 gene. CATCH and a similar method, CISMR [4], involve pulse-gel electrophoresis step which makes these methods labour and time-consuming. Yet, because of the amplification step, the listed methods of TGSeq do not allow simultaneous sequencing and profiling of methyl-cytosine bases, a key player in gene transcription regulation.

Recently, a new method called nCATS for target sequencing of native DNA molecules has been proposed. With no PCR amplification step nCATS can generate data suitable for both sequencing and profiling of DNA methylation of target genes [5]. The method is based on the selective Oxford Nanopore sequencing of DNA fragments released after Cas9-mediated cleavage of total genomic DNA. In contrast to CATCH, in nCATS no gel-electrophoresis is required to separate Cas9-cleaved fragments from non-target genomic fragments. Instead, to deplete non-target DNA nCATS uses calf intestinal alkaline phosphate (CIP) enzyme. CIP performs 5′ dephosphorylation of genomic DNA fragments before Cas9 digestion step making DNA ends unsuitable for ligation of sequencing adapters. In turn, subsequent Cas9/sgRNA treatment introduces double-strand breaks that together with dA-tailing make DNA ends suitable for adapter ligation. Thus, adapters are mostly ligated to the Cas9-cleaved DNA fragments resulted in higher chances for target regions to be sequenced by nanopore. By nCATS authors were able to rich up to 400× coverage of 18 Kb target region using MinION sequencer which was sufficient for identification of single nucleotide changes, structural variation and evaluating DNA methylation in human [5].

It is worth noting that nCATS does not include PCR-amplification therefore obtained raw signal nanopore data is suited for cytosine methylation identification using one of the available algorithms [6]. Methylation profiling of targeted genes followed by nCATS have been carried out for mammals [5,7]. An algorithm for methylation-calling from raw-signal nanopore data with plant-specific trained models has recently been released providing the foundation for the application of nCATS-based methylation profiling in plants [8]. An effort to apply nCATS for the sequencing of plant genes was made [9]. Using 4 sgRNAs authors sequenced ~7.8 Kb *MYB10* locus of apple (*Malus × domestica*) achieving > 100× target coverage and performed haplotype phasing. It should be noted that, the apple genome is smaller (730.10 Mb/1 C for *Malus × domestica* [10]) than many other agronomical important species including grasses (Poaceae) for which genome size varies from 276 Mb/1 C to 20,825 Mb/1 C (https://cvalues.science.kew.org/, accessed on 2 November 2021). For example, the genome sizes of wheat (*Triticum aestivum*) and rye (*Secale cereale*) are 16,954 and 8624 Mb/1 C [11], respectively. Therefore, a question raised is whether nCATS is efficient for target gene sequencing for plant species with big genome size. In addition, whether the nCATS is applicable to sequence several plant genes (multiplexing) in one run. Finally, gene methylation profiling using nCATS data has not been exploited so far in plants. Here, we aimed to address these questions by sequencing of full-length glutenin genes (*Glu-Ax*, *Glu-1Bx* and *Glu-1By*) and their promoters in hexaploid triticale (X *Triticosecale*, AABBRR, genome size is 24 Gb [12]), a human-made crop obtained from hybridization of wheat and rye. A choice of target genes is not random as the glutenin genes are important for triticale improvement [13]. Yet, these genes are difficult to assemble from short reads because of ~90% of the gene sequence contains low-complexity, repetitive elements [14].

Here, we demonstrated that nCATS can be used as a potential tool for TGSeq in plants with a big genome. The obtained low sequencing depth was sufficient for various tasks including detection of InDels and single-nucleotide variations (SNPs), read phasing and methylation profiling. Using nCATS data, we demonstrated that glutenin genes possess gene-body methylation with hypermethylated CDS part and hypomethylated promoter regions. While further improvement is needed, our proof-of-concept work shows the potential of nCATS approach for sequencing of target genes in plants with big genome size.

## 2. Results

**Figure 1:**
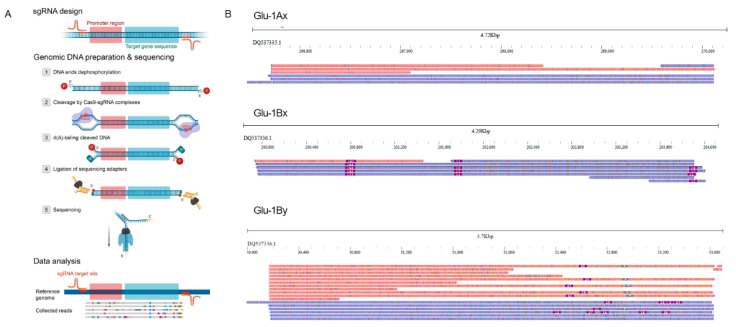
(**A**) A schematic view of nCATS pipeline used in this study. (**B**) The jbrowse2 snapshots showing the alignment of the reads to the target sequences. Blue and red colors correspond to the reads mapped on positive and negative strands, respectively.

For nCATS sequencing of *Glu-1Ax*, *Glu-1Bx* and *Glu-1By* glutenin genes we designed two pairs of sgRNAs for each gene (Figure 1A). Because the glutenin genes are not correctly assembled in the wheat genome, for sgRNA design we used publicly available sequences of these genes from BAC clones while wheat and rye genome sequences were involved in ‘off-target’ site prediction (see Section 4). The target regions included the coding sequence of Glu genes and promoter regions. The expected sequence lengths for the target regions were 3.4 Kb, 5.1 Kb and 3.6 Kb for *Glu-1Ax*, *Glu-1Bx* and *Glu-1By*, respectively. We performed two runs of nCATS on MinION sequencer with a mixture of all 12 sgRNAs. For further analysis, the reads from these two runs were merged into one fastq file. In total, we obtained 120,681 high-quality (Qscore > 8, N50 = 3.1 Kb) nanopore reads. We calculated the number of on-target reads by similarity search of read sequences against reference glutenin genes and found 7, 8 and 17 reads for *Glu-1Ax*, *Glu-1Bx* and *Glu-1By* loci, respectively. Consequently, ~0.03% obtained nanopore reads were on-target reads. Although the overall number of on-target reads were quite low, the enrichment rate for the three target genes varied from ~200× to ~645× (Table 1) based on the triticale genome size (24 Gb, [12]) and total length of the obtained Nanopore reads (~547 Mb, 0.02× triticale genome coverage).

Most of the obtained reads covered full-length gene sequence (Figure 1) providing useful information for structural variation identification of the glutenin gene variants of the triticale line used for sequencing (L8665). Indeed, we were able to easily uncover two insertions present in the *Glu-1Bx* gene. One insertion of ~180 bp was located in the promoter region while the second insertion of 12bp was located in the coding region of *Glu-1Bx* (Figure 1B). To validate these results, we designed primer pairs and performed PCR with genomic DNA of L8665 triticale line and wheat cv. Chinese Spring. The PCR results and Sanger sequencing also proved the presence of the insertions in *Glu-1Bx* variants in L8665 line (Supplementary Figure S1). Comparing the sequence of L8665 *Glu-1Bx* variant with the previously sequenced glutenin genes showed that our triticale line carries *Glu-1Bx14* allele. These results demonstrate that even low (5×) coverage of the target gene by nanopore reads allows sophisticated identification of InDels and PCR marker design.

**Figure 2:**
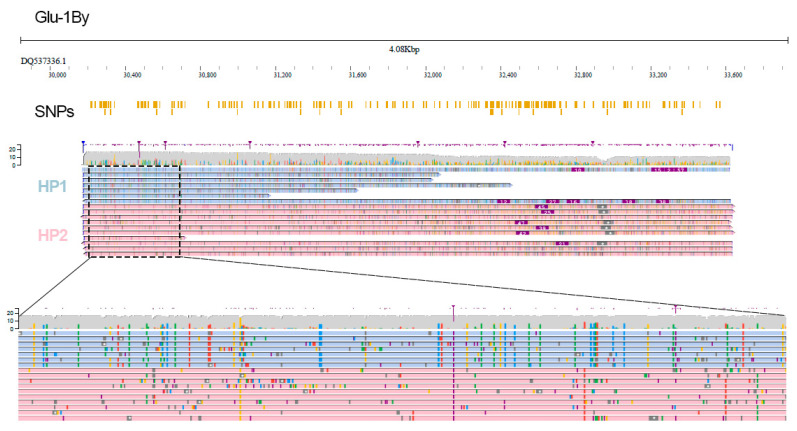
Visual representation of high-confidence SNP variants detected by NanoCaller pipeline in the nanopore data and read phasing into parental alleles of *Glu-1By* gene (light blue and pink reads correspond to HP1 and HP2 alleles, respectively) established by WhatsHap. SNPs track shows all high-confidence SNPs detected by NanoCaller.

We next tested SNP identification which was possible for the *Glu-1By* gene having the highest sequencing depth (~15×). Overall, we identified 222 SNPs distinguishing the *Glu-1By* gene variant of L8665 from the reference and then applied WhatsHap to assign reads to haplotypes based on SNPs detected in nanopore data. Using these SNPs we successfully phased the reads (Figure 2). We generated an individual haplotype sequence of *Glu-1By* and performed a similarity search using BLAST followed by phylogenetic analysis restricted to a unique part of glutenin CDS (~300 bp). This analysis revealed that HP1 and HP2 alleles are clustered with known *Glu-1By* genes of wheat (Supplementary Figure S2) but they are not fully similar to the known *Glu-1By* alleles suggesting that they may be new variants of the *Glu-1By* gene.

**Figure 3:**
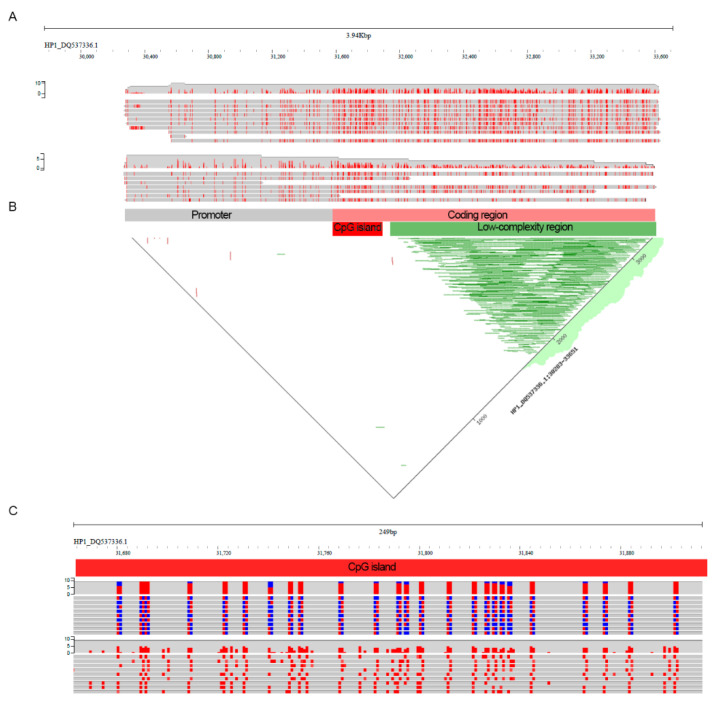
Methylation profile of the *Glu-1By* gene. (**A**) Per-read methylation distribution of the *Glu-1By* gene obtained after DeepSignal-plant methylation calling. For visualization, the MM tag was added to alignments in the bam file using DeepS2bam_converter. The visualization was performed in jbrowse2 installed on the local server. (**B**) A dot plot showing repetitive parts of the *Glu-1By* gene (green lines). (**C**) Zoomed-in part of the *Glu-1By* gene with long CpG island. Methylated (red) and unmethylated (blue) cytosine of the CpG context are shown on the top panel. All and only methylated cytosines are shown on the bottom panel.

Nanopore data provides unique information on DNA methylation and we were interested to explore cytosine methylation (meC) of the full-length glutenin genes. For this, we performed methylation calling from raw nanopore reads using a recently published algorithm (DeepSignal-plant). Unfortunately, there are no direct ways to represent read-level methylation plots for DeepSignal-plant data therefore we designed the custom script, DeepS2bam_converter, to add MM tag to each unique alignment in the bam file. This allowed us to display methylation information from nanopore sequencing in per-read mode (Figure 3A). While the read depth for *Glu-1Ax* and *Glu-1By* is low we were able to compare the methylation profiles for the three glutenin genes using per-read methylation display (Supplementary Figure S3). In general, the methylation profile was similar between the three genes with low methylated promoter region and highly methylated coding sequence. These results show that glutenin genes have a strong pattern of gene-body methylation. We further analyzed the methylation profile of the *Glu-1By* gene as it has higher read coverage. The two alleles of the Glu1-By gene have a similar distribution of meC marks (Figure 3A).

Because glutenin genes have a long low-complexity region in the protein-coding part we asked whether this region may interfere with the methylation identification. To check this assumption, we identified this region in the *Glu-1By* gene using a dot plot (Figure 3B) and focused on a part of the glutenin coding sequence upstream of the low complexity region. We revealed that this ‘unique’ region of *Glu-1By* is consistently methylated across the reads (Figure 3B). Analysis of CpG distribution revealed one CpG island with >200 bp length located in the unique part of the glutenin coding sequence. This region exhibits strong methylation across all nanopore reads (Figure 3C). Taken together, our results showed that in leaf tissue glutenin genes possess gene-body methylation marks with heavily methylated CpG islands in a non-repetitive part of the coding region.

## 3. Discussion

Simultaneous sequencing and epigenetic profiling of plant genes and their promoters are attractive because the obtained data can be used to investigate the variation of genes and their regulatory sequences on genetic and epigenetic levels. In turn, it may provide a foundation for the study of epigenetic control of spatiotemporal gene expression patterns, a poorly studied field especially in plants with big and complex genomes such as wheat and triticale. The previously developed method, nCATS, was efficient for target sequencing in human [5] and plants with relatively small genomes [9,15]. But in triticale and wheat, a target gene occupies only a millionth part of a genome (e.g., 5 Kb gene is 1/4,800,000 part of the triticale genome). Therefore, the application of nCATS for these species resembles ‘searching for a needle in a haystack. Here, using triticale and glutenin genes as targets we demonstrated that nCATS is a useful method although low sequencing depth should be expected, and more flow cells are required. Previously, we applied Cas9-targeted sequencing for *Arabidopsis thaliana*, a plant with a tiny genome (157 Mb/1 C, [16]) and achieved 40× coverage of target sequence after 4 h of MinION sequencing [15]. Also, sequencing of ~7.8 Kb *MYB10* locus of apple (730.10 Mb/1 C for *Malus × domestica* [10]) by nCATS resulted in >100× target coverage. Based on this, we suppose that the relatively low efficiency of nCATS in triticale is a direct consequence of the big genome (genome size is 24 Gb/1 C [12]). To make nCATS more cost-effective for plants with big genome size in the future, a combination of multiple sgRNAs and a higher number of genes can be applied. Indeed, during nanopore sequencing of nCATS DNA library, only a few percent of pores are sequencing (up to 5% in our hands) and including more target genes and sgRNAs may increase the sequencing efficiency [5]. Another option is to perform enrichment of target DNA fragments by, for example, purification from the gel as it was originally proposed in the CATCH method [3]. In addition, the improvement of high-molecular-weight DNA isolation and size-selection protocols is a simple but crucial strategy toward increasing nCATS output.

The results of our work provide new biological insights into the glutenin gene organization. It was known that transcription regulation of prolamins is achieved by binding transcription factors to the motifs of the promoter [17,18]. Also, it was shown that DNA methylation may play a key role in the expression of gluten proteins [19,20]. Methylation of the promoter region of glutenin genes established by bisulfite sequencing showed an increased meC level in flag leaves compared to the developing grain [20]. However, the methylation profile of the coding region of glutenin genes has not been studied so far. Here, taking advantage of direct DNA nanopore sequencing we showed that the promoter of glutenin genes is much lower methylated than the coding region. This methylation distribution along transcribed part of glutenin genes resembles ‘gene body’ methylation (gbM). GbM is often an attribute of housekeeping, constitutively expressed and conserved genes [21]. However, glutenin genes do not fit these characteristics as they have endosperm-specific expression patterns and demonstrate high variability. The latter is also supported by our SNP analysis of *Glu-1By* gene alleles which revealed >100 SNPs differentiated two alleles. It should be noted that the analysis of the DNA methylation profile of the genes with the endosperm-preferred expression revealed that these genes are prone to have increased gbM in rice [22] which is in concordance with our results. While we have not analyzed the differences in methylation profile between leaves and developing seeds it would be interesting to do in the future to assess the role of gbM in the regulation of the transcription program of glutenin genes.

In summary, our work demonstrates the potential of the nCATS approach for sequencing of target genes in plants with big genome size and provides novel information on the methylation profile of glutenin genes in triticale.

## 4. Materials and Methods

### 4.1. Plant Material and DNA Isolation

For this study, the spring triticale line “L8665” obtained from the Department of Genetics, Russian State Agrarian University, was used. Seeds of this line were germinated at room temperature on wet filter paper disks. High molecular weight DNA was isolated from 200–500 mg material that was homogenized in liquid nitrogen. DNA isolation was done according to the published protocol (https://www.protocols.io/view/plant-dna-extraction-and-preparation-for389-ont-seque-bcvyiw7w, accessed on 4 September 2021). Isolated DNA was used for size-selection of the large DNA fragments by SRE or XL Short Read Eliminator Kits (Circulomics, Baltimore, MD, USA) according to the manufacturer’s instructions. The concentration and quality of the isolated DNA were assessed by NanoDrop One UV-Vis Spectrophotometer (Thermo Scientific, Waltham, MA, USA) and Quantus Fluorometer (Promega, Madison, WA, USA) using a DNA QuantiFluor ONE dsDNA System (Promega, Madison, WA, USA). For sequencing, DNA with A260/A280 ~1.8 and A260/A230 ~2.0 according to NanoDrop and with equal concentrations according to Nanodrop and Quantus was used.

### 4.2. gRNA Design and In Vitro Transcription

gRNAs were designed on BAC clone sequences of the three glutenin genes: DQ537335.1 (NCBI accession number) for *Glu-1Ax* and DQ537336.1 for *Glu-1Bx* and *Glu-1By*. gRNAs were designed by CRISPRdirect (https://crispr.dbcls.jp/, accessed on 3 September 2021 [23]) and FlashFry [24]. Additionally, we aligned the known glutenin alleles from NCBI and selected gRNAs with the maximum number of potential target alleles. Four gRNAs (2 forward and 2 reverse) were designed for each gene. SgRNAs for nCATS were produced by in vitro transcription from DNA templates containing T7 promoter according to [15]. The templates were assembled from two oligos, gRNA—specific (Table 2, GGATCCTAATACGACTCACTATAGGxxxxxxxxxxxxxxxxxxxGTTTTAGAGCTAGAA, where xxx is gRNA sequence) and universal (CRISPR_R: AAAAAAGCACCGACTCGGTGCCACTTTTTCAAGTTGATAACGGACTAGCCTTATTTTAACTTGCTATTTCTAGCTCT). All oligonucleotides were ordered in Evrogen (Moscow, Russia).

The sgRNA synthesis was carried out according to the previous protocol [15]. The concentration and quality of prepared sgRNAs were estimated by Nanodrop (Thermo Scientific, Waltham, MA, USA), Qubit (Thermo Scientific, Waltham, MA, USA) and gel electrophoresis in 2% agarose gel. (Table 2).

### 4.3. nCATS Library Preparation

nCATS library preparation for nanopore sequencing was carried out according to the previously published protocols [5,15] and using SQK-LSK109 (Oxford Nanopore Technologies, Oxford, UK). Briefly, RNP assembly was carried out using 200 ng of each sgRNA and 8 pmol Cas9 protein (Biolabmix, Novosibirsk, Russia). ~3 µg of genomic DNA was cleaved by the RNP complexes for each library. After cleavage, dA-tailing and adapter ligation the samples were diluted by 1 volume of TE buffer, purified by 0.3 volume of AMPure XP Beads (Beckman Coulter, catalogue no. A63881, Brea, CA, USA) and washed twice by SFB buffer (Oxford Nanopore Technologies, catalogue no. SQK-LSK109).

### 4.4. Nanopore Sequencing and Basecalling

Sequencing was performed by MinION equipped with R9.4.1 flow cell. The sequencing process was operated by MinKNOW software v19.12.5 (Oxford Nanopore Technologies, Oxford, UK). Basecalling was carried out by Guppy v5.0.14 5 (Oxford Nanopore Technologies, Oxford, UK).

### 4.5. SNP Calling and Phasing

The obtained nanopore reads were aligned to the full-length sequences of BAC clones with target sequences using minimap2 software [25] with the following parameters: -ax map-ont-t 100. The obtained sam file was converted to bam format, sorted and indexed using SAMtools [26]. For SNP calling and read phasing Nanocaller pipeline was exploited [27] with--enable_whatshap-keep_bam flags to allow the bam file modification. To reconstruct sequences of *Glu-1By* alleles ‘bcftools consensus’ command from bcftools [28] was applied. Phasing was performed by WhatsHap [29] as a part of the NanoCaller pipeline.

### 4.6. Methylation Calling and Visualization

Methylation calling using nanopore raw read data was done by DeepSignal-plant [8] software. For this, reads were basecalled by Guppy (v5.0.14) and converter to single fast5 files by multi_to_single_fast5 command from ont_fast5_api package (https://github.com/nanoporetech/ont_fast5_api, accessed on 2 September 2021). Then reads and fast5 files were preprocessed by tombo preprocess (default parameters) and tombo resquiggle (default parameters) commands. After this, deepsignal_plant call_mods command was applied to call methylation using model model.dp2.CNN.arabnrice2-1_120m_R9.4plus_tem.bn13_sn16.both_bilstm.epoch6.ckpt. For per-read methylation visualization, the bam file was modified (MM tag was added) using custom made script DeepS2bam_converter (https://github.com/Kirovez/DeepS2bam_converter, accessed on 12 September 2021). Then the read alignments and methylated cytosine were visualized by JBrowse2 [30] using colouring by modifications option.

### 4.7. PCR Validation of the Insertion in Glu-1Bx Gene

To validate 2 InDels (180 bp and 12 bp) located in the *Glu-1Bx* gene and identified by nCATS, the primers listed in Table 3 were used. PCR was performed with Encyclo DNA polymerase (Evrogen, Moscow, Russia) according to the manufacture’s instruction. The PCR conditions were 94 °C for 1 min; 35 cycles of 94 °C for 1 min, 60 °C for 1 min, and 72 °C for 1 min; and a final elongation of 72 °C for 3 min.

### 4.8. Phylogenetic Tree Construction

To construct the phylogenetic tree with the known glutenin genes and two *Glu-1By* alleles (HP1 and HP2 alleles) reconstructed from our data, we performed a similarity search using BLAST. For this, only the non-repetitive part of *Glu-1By* CDS (~300 bp) was exploited. The sequences with >80% similarity were extracted from BLAST search results using the ‘Download aligned sequences’ function. The obtained sequences were imported to the NGPhylogeny.fr online tool (https://ngphylogeny.fr/, accessed on 5 October 2021 [31]) and the phylogenetic tree was constructed using the default mode.

### 4.9. Visualization and Data Analysis

The read alignments were visualized in JBrowse2 [30]. The dot plot was constructed by YASS [32] (https://bioinfo.lifl.fr/cgi-bin/yass/, accessed on 5 October 2021).

## Figures and Tables

**Table 1:** General information on nCATS sequencing results.

Locus	Number of On Target Reads	Enrichment Rate
*Glu-1Bx* DQ537336.1:199,854..204,146	8	~200×
*Glu-1By* DQ537336.1:29,265..35,054	17	~645×
*Glu-1Ax* DQ537335.1:265,694..270,243	7	~200×

**Table 2:** gRNAs designed for each target gene.

Locus	gRNA Sequence
*Glu-1Bx* DQ537336.1:199,854..204,146	F1: AAAACGTCCATGCATAAGTA; F2: ATTACATGTAGCCACCGACA; R1: TCACGTTTATTGTATAGCTA; R2: CAGAGAGTTCTATCACTGCC
*Glu-1By* DQ537336.1:29,265..35,054	F1: GGGCCCTGTGCGGTTCGCAC; F2: CCTGGATTATGTTGGACGAT; R1: CCCTCCATCCGACACATTAT; R2: TGCTCTGTGTTAACATGGTA
*Glu-1Ax* DQ537335.1:265,694..270,243	F1: GCAACGATTATGGGGCTGCA; F2: CTCCCTCATGAGTTGTATGC; R1: ATGCGTCGCCGCCCTCTAGC; R2: TGCTCCGCGCTAACATGGTA

**Table 3:** Primers used for InDel validations in the *Glu-1Bx* sequence.

Primer Id	Primer Sequences	Insertion Name
Glu_x_prom F	caaccatgcatagaagaaagctc	Insertion 180
Glu_x_prom R	ccttcttggggtttggcaga	
BxUnique1_350F	ccctgctgcgaagaagttac	Insertion 12
BxUnique1_350R	tggcctggatagtatgacccctg	

## Data Availability

Nanopore data produced for this study are available in Sequence Read Archive (SRA) NCBI under Bioproject Accession PRJNA783195.

## Supplementary Materials

The following supporting information can be downloaded at: https://www.mdpi.com/article/10.3390/plants11010005/s1, Figure S1: PCR results with primer pairs flanking the InDels of *Glu-1Bx* variant, Figure S2: Neighbor-joining phylogenetic tree constructed after multiple alignment of unique parts of CDS region of HP1 and HP2 alleles of *Glu-1By* gene, Figure S3: Per-read methylation distribution of the three glutenin genes obtained after DeepSignal-plant methylation calling.
