# Supplementary material for: Searching for a Needle in a Haystack: Cas9-Targeted Nanopore Sequencing and DNA Methylation Profiling of Full-Length Glutenin Genes in a Big Cereal Genome
Source: Plants (Basel). 2021 Dec 21;11(1):5. doi: 10.3390/plants11010005 (PMC8746966; doi:10.3390/plants11010005)
Supplement: Supplementary file 1 [file plants-11-00005-s001.zip › plants-1502680-supplementary.pdf]

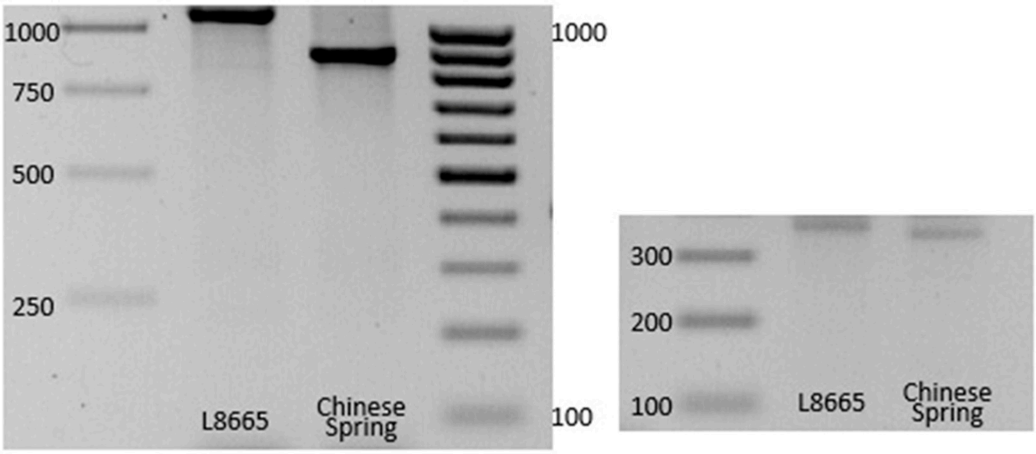

Supplementary Figure S1: PCR results with primer pairs flanking the InDels of Glu-1Bx variant: 180bp (left) and 12bp (right). PCR was conducted with genomic DNA of wheat cv. Chinese Spring (no insertions) and triticale cv. L8665 (insertions are present).

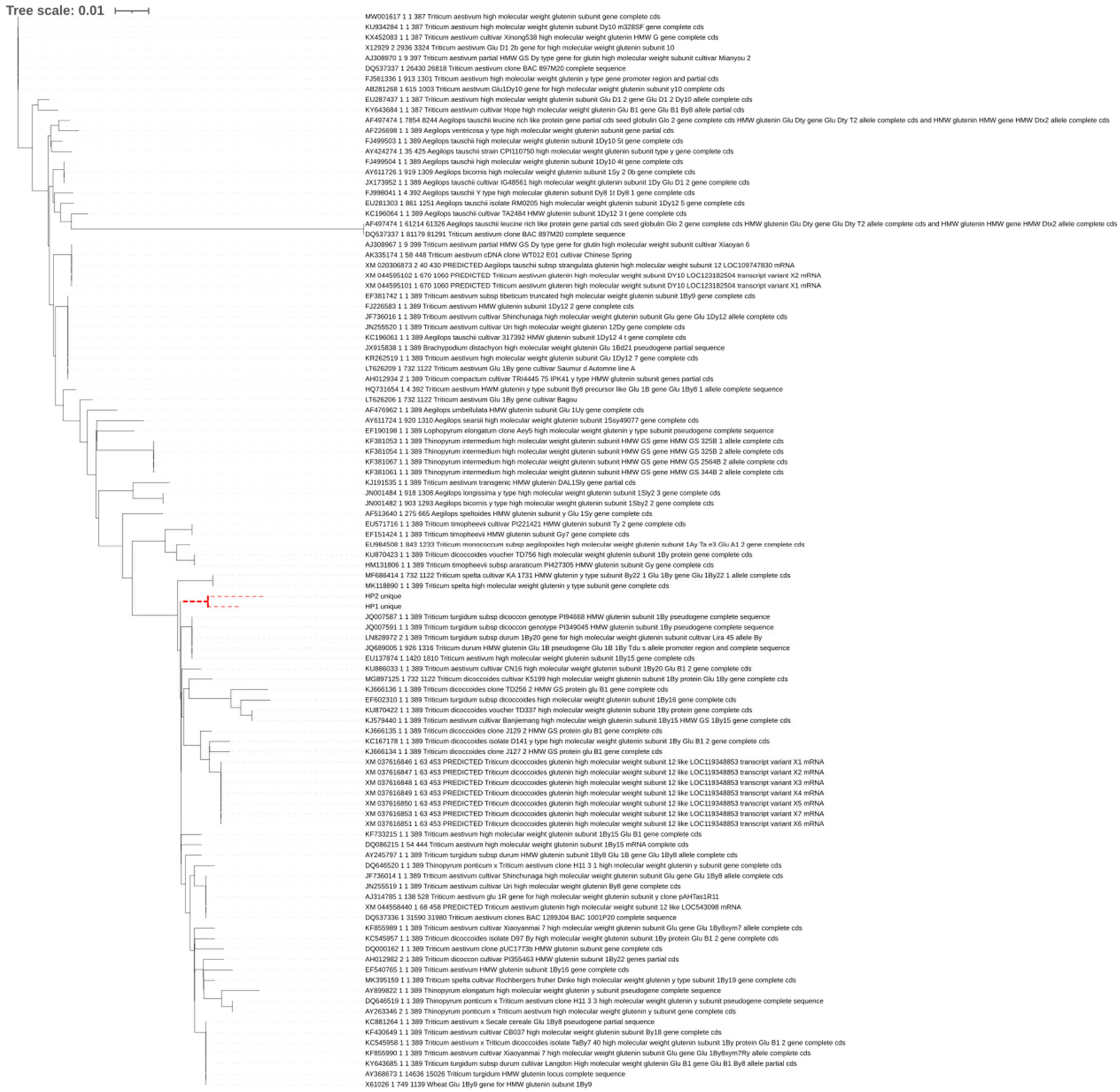

Supplementary Figure S2: Neighbor-joining phylogenetic tree constructed after multiple alignment of unique parts of CDS region of HP1 and HP2 alleles of Glu-1By gene.

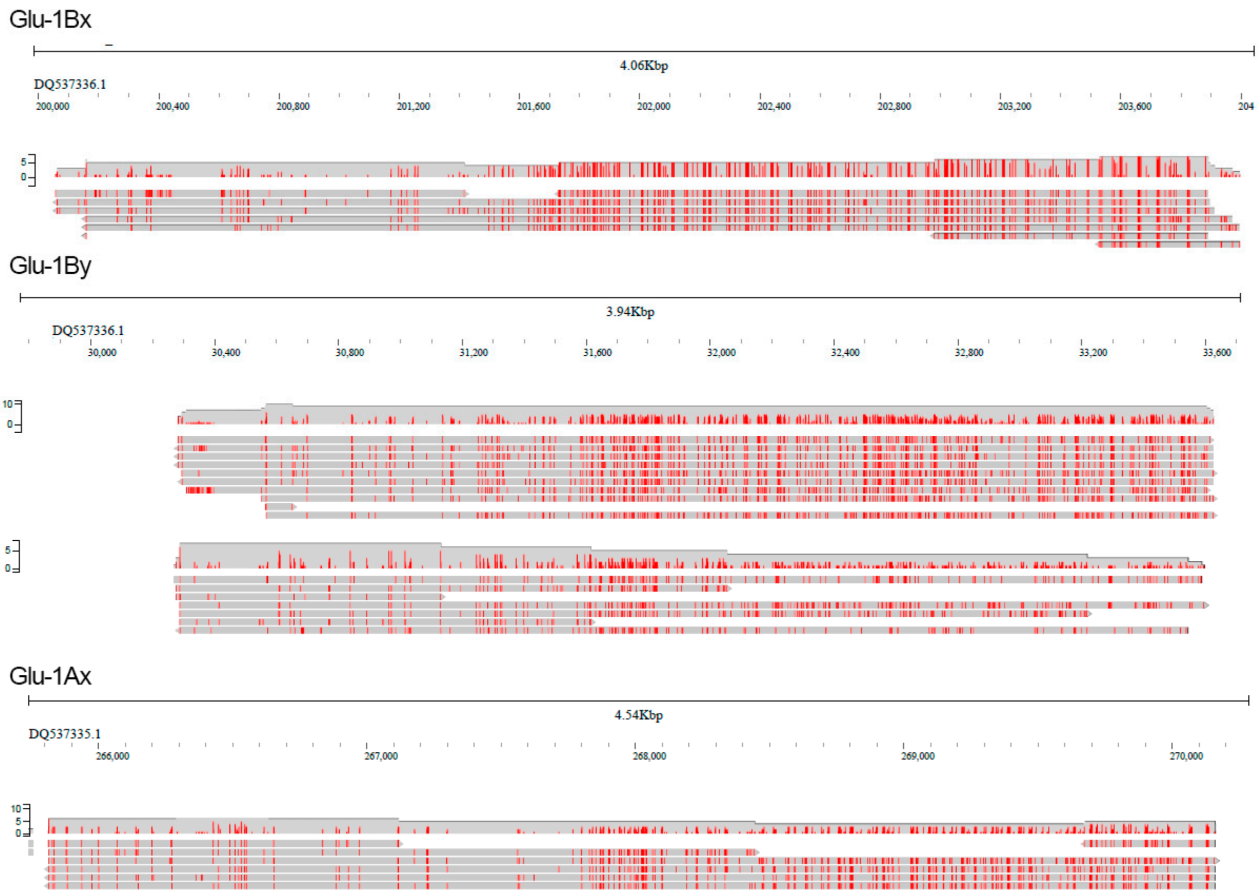

Supplementary Figure S3: Per-read methylation distribution of the three glutenin genes obtained after DeepSignal-plant methylation calling. For visualization the MM tag was added to alignments in the bam file using DeepS2bam_converter. The visualization was performed in JBrowse2 (https://jbrowse.org/jb2/) installed on the local server.
